# Gout flares following acute stroke: a single-center cohort and a systematic review/meta-analysis

**DOI:** 10.1186/s42466-025-00424-w

**Published:** 2025-10-15

**Authors:** Evangelos Panagiotopoulos, Vasiliki Kotsali-Peteinelli, Georgia Papagiannopoulou, Aikaterini Theodorou, Maria Chondrogianni, Eleni Bakola, Lina Palaiodimou, Klearchos Psychogios, Odysseas Kargiotis, Apostolos Safouris, Panagiota-Eleni Tsalouchidou, Annerose Mengel, Ulf Ziemann, Christos Krogias, Georgios Tsivgoulis, Maria-Ioanna Stefanou

**Affiliations:** 1 Second Department of Neurology, School of Medicine, “Attikon” University Hospital, National and Kapodistrian University of Athens, Athens, Greece; 2 Stroke Unit, Metropolitan Hospital, Piraeus, Greece; 3 Department of Neurology, School of Medicine, University Hospital of Patras, Patras, Greece; 4 Department of Neurology & Stroke, Eberhard-Karls University of Tübingen, Tübingen, Germany; 5 Hertie Institute for Clinical Brain Research, Eberhard-Karls University of Tübingen, Tübingen, Germany; 6 Department of Neurology, St. Josef-Hospital, Ruhr University, Bochum, Germany

**Keywords:** Gout, Gouty arthritis, Hyperuricemia, Uric acid, Stroke, Colchicine

## Abstract

**Background:**

With the global prevalence of hyperuricemia and gout rising, accumulating evidence has linked acute gout flares to a transient rise in major adverse cardiovascular events, including myocardial infarction and stroke. However, the reverse association, whether acute stroke is associated with an increased incidence of gout flares, has been inadequately investigated. The aim of this single-center cohort study, coupled with a systematic review and meta-analysis, was to evaluate the incidence and characteristics of gout flares in the early post-stroke period.

**Methods:**

A systematic review and meta-analysis of published studies was conducted, incorporating data from a cohort of acute stroke patients admitted to a tertiary care stroke center. Eligible studies reported in-hospital gout flares among patients with acute ischemic or hemorrhagic stroke. Pooled estimates were calculated using random-effects models. The systematic review was pre-registered in PROSPERO (CRD420251061747).

**Results:**

We identified three studies (one involving only acute ischemic stroke, two including both ischemic and hemorrhagic stroke), encompassing a total of 13,722 acute stroke patients, including our institutional cohort. The pooled incidence of in-hospital gout flares was 4% (95% CI, 2–6%; I²=88.1%). The pooled mean time to flare onset was 6.3 days post-stroke (95% CI, 4.09–8.44; I²=96.6%), and 64% of flares involved the paretic limb (95% CI, 33–90%; I²=62.1%). Among flare patients, 85% were male (95% CI, 40–100%; I²=84.5%), 97% had ischemic stroke (95% CI, 94–99%; I²=0%), and 61% were newly diagnosed with gout or hyperuricemia during hospitalization (95% CI, 48–73%; I²=0%). In our cohort, 50% and 12.5% of flare patients presented with delirium and aphasia, respectively; all patients received colchicine treatment with complete symptom resolution.

**Conclusions:**

Gout flares occur in one out of every 25 acute stroke patients, particularly in men presenting with acute ischemic stroke within the first week after symptom onset, and frequently involve the paretic limb. In over half of cases, the flare uncovers previously undiagnosed gout or hyperuricemia, emphasizing the need for systematic evaluation, particularly in patients with delirium or impaired communication, where diagnosis may be delayed. Early colchicine initiation warrants consideration given its anti-inflammatory effects and potential to reduce stroke recurrence.

**Supplementary Information:**

The online version contains supplementary material available at 10.1186/s42466-025-00424-w.

## Background

Gout, or uric arthritis, comprises a chronic inflammatory arthritis driven by monosodium urate crystal deposition in joints and affects nearly 10% of patients with history of stroke [1, 2]. Acute gout episodes, defined as flares, present with severe joint inflammation, including pain, erythema, swelling, and elevated temperature [3]. Although prior research has established that both chronic and acute gout increase the risk of stroke [4–6], the reverse association, whether acute stroke precipitates an increased risk of gout flares, has received limited attention.

The pathophysiologic overlap between gout and stroke involves shared mechanisms of vascular inflammation. In chronic gout, low-grade inflammation mediated by interleukin-1β (IL-1β), IL-6, and tumor necrosis factor-α (TNF-α) promotes endothelial dysfunction, oxidative stress, and atherogenesis [7–9]. During acute flares, monosodium urate crystals activate the NLRP3 inflammasome, triggering IL-1β release, mitochondrial dysfunction, and plaque destabilization [7]. Similarly, acute stroke initiates aberrant inflammatory cascades, encompassing sympathetic activation, blood–brain barrier disruption, and systemic cytokine elevation [10]. Stroke has also been associated with transient hyperuricemia, likely secondary to oxidative stress–driven purine metabolism [11]. These findings suggest a bidirectional inflammatory link in which gout increases stroke risk, and acute stroke may precipitate gout flares.

Recognizing this overlap is crucial in guiding anti-inflammatory strategies with relevance across both conditions. Colchicine, a standard therapy for gout, is being evaluated for secondary stroke prevention [12–14]. A recent meta-analysis of randomized-controlled clinical trials (RCTs), comprising 14,934 patients with prior stroke or coronary artery disease demonstrated that colchicine reduced ischemic stroke risk by 27% compared with placebo (Risk Ratio [RR]: 0.73; 95% Confidence Interval [CI]: 0.58–0.90), without increasing serious adverse events [12].

Despite the growing pathophysiological rationale supporting the association between gout and stroke, clinical recognition of gout flares in stroke patients remains limited. Up to 30% of patients with acute stroke develop post-stroke delirium [15–18], and 17% present with aphasia [19], both conditions compromising effective pain communication. Inflammation during gout flares may also contribute to delirium [20], while sensory deficits in paretic limbs may mask nociceptive signals, further delaying diagnosis [21]. Unrecognized flares may lead to persistent hyperuricemia, associated with a 1.4-fold increased risk of stroke recurrence and a 1.5-fold increase in stroke mortality [22].

In view of the former considerations, a systematic review and meta-analysis of published studies on gout flares in patients with acute stroke (ischemic or hemorrhagic) was conducted, incorporating data from our single-center cohort. Our aim was to investigate the incidence and clinical characteristics of these events and to identify strategies for improved recognition and management.

## Methods

### Data availability

All datasets analyzed during the present study are included in this article and its Supplemental Material.

### Single-center cohort

Patients with acute stroke (ischemic or hemorrhagic), admitted to the Comprehensive Stroke Unit of a tertiary academic hospital (Second Department of Neurology, Attikon University Hospital, Athens, Greece), and who developed gout flares during hospitalization, were prospectively registered over a 12-month period (February 2024 – February 2025). Institutional review board approval was obtained from the Ethics Committee of Attikon University Hospital (decision number EBD 444/13-06-2024). All patients or their legal representatives provided written informed consent to participate in the study, which was conducted in accordance with the Declaration of Helsinki. The institutional cohort was reported in accordance with the STROBE (Strengthening the Reporting of Observational Studies in Epidemiology) statement [23].

The diagnosis of gout flares was based on a combination of clinical evaluation and the ACR/EULAR (American College of Rheumatology / European League Against Rheumatism) 2015 Gout Classification Criteria [24]. All patients underwent a comprehensive diagnostic work-up to determine the etiology of stroke, in accordance with current practice guidelines [25, 26].

### Meta-analysis

#### Standard protocol approvals and registrations

The present meta-analysis was conducted in accordance with the Preferred Reporting Items for Systematic Reviews and Meta-Analyses (PRISMA) guidelines [27] and the pre-specified protocol was registered in PROSPERO (CRD420251061747). Ethical board approval was not required for the systematic review and meta-analysis, as per study design.

#### Data sources and database searches

A systematic literature search was conducted independently by three reviewers (EP, VKP, GP) to identify eligible studies reporting gout flares in patients with acute stroke - either ischemic or hemorrhagic - from inception of each database to May 29, 2025. We searched PubMed and Scopus using the following search strings: “gout”, “gouty arthritis”, “uric arthritis”, “gout flares”, “gout attack”, and “stroke”. The complete search algorithms are provided in the Supplemental Material. No language or other restrictions were applied. The three independent reviewers (EP, VKP, GP) reached a consensus on the final screening list, with any disagreements resolved after discussion with the corresponding author (MIS).

### Study selection

Relevant studies were screened systematically by three independent reviewers (EP, VKP, GP) based on titles and abstracts. The following inclusion criteria were applied: observational studies or RCTs involving patients admitted with acute stroke who experienced gout flares during hospitalization following the index stroke. Studies were excluded if they met any of the following criteria: (i) narrative reviews, systematic reviews, meta-analyses, case reports and case series, commentaries, non-peer-reviewed studies, pre-prints, or conference abstracts; (ii) studies reporting clinical presentations of gout in stroke patients without documented gout flares based on the ACR/EULAR2015 Gout Classification Criteria [24]; or (iii) studies involving patients with gout flares preceding the index stroke.

To confirm eligibility, three independent reviewers (EP, VKP, AT) assessed the full-text articles of all potentially eligible studies. Studies not meeting the predefined inclusion criteria were excluded. The final study selection was also performed by these three reviewers independently (EP, VKP, AT), with any disagreements resolved through consensus in consultation with the corresponding author (MIS).

### Data extraction

The following data were extracted for analysis: study name, first author, year of publication, country, study design, total number of patients, type of stroke, sex distribution, history of hyperuricemia or gout, mean time from index stroke to flare onset, flare localization by limb involvement (paretic versus non-paretic), and treatment administered during the flare.

### Outcomes

An aggregate data meta-analysis was conducted to pool estimates for all outcomes of interest. The primary outcome was the cumulative incidence of in-hospital gout flares. Secondary outcomes comprised the mean time from index stroke to flare onset and the localization of gout flares by limb involvement (paretic versus non-paretic). Additional outcomes encompassed sex distribution, the proportion of patients with ischemic stroke among all patients presenting with gout flares, the number of patients with a first-time diagnosis of gout or hyperuricemia during hospitalization, and the treatment administered during the flare.

### Study quality and assessment of publication bias

Risk of bias for each included study was assessed using the Cochrane Collaboration’s Risk of Bias in Non-randomized Studies of Interventions (ROBINS-I) tool for observational studies [28]. Quality control and bias assessment were conducted independently by four reviewers (EP, VKP, MC, EB), with disagreements resolved through discussion with the corresponding author (MIS). Publication bias was not formally assessed, as fewer than ten studies were included in the meta-analysis, a threshold below which funnel plot asymmetry tests are considered unreliable [29].

### Statistical analysis

All statistical analyses were conducted using R–software (packages: meta and metafor). Meta-analyses were conducted using a random-effects model according to the DerSimonian and Laird method [30]. Pooled estimates with corresponding 95% CIs were calculated for all outcomes. A two-tailed *p*-value of ≤ 0.05 was considered statistically significant. Heterogeneity between studies was assessed with the Cochran Q and I^2^ statistics [31]. I^2^ values exceeding 50% and 75% were considered indicative of substantial and considerable heterogeneity, respectively. A *p*-value < 0.10 for the Cochran’s Q test indicated heterogeneity.

For the single-center cohort, continuous variables were expressed as mean ± standard deviation (SD), and categorical variables as counts and percentages. Fisher’s exact test was applied for comparisons of categorical variables with small expected frequencies. Associations between baseline characteristics and in-hospital gout flares were assessed using logistic regression to estimate odds ratios (OR) with 95% confidence intervals (CI). For zero-cell counts, the Haldane–Anscombe correction was applied. The independent variables - age, sex, NIHSS score, hypertension, diabetes, smoking, atrial fibrillation, dyslipidemia, and alcohol use - were examined in separate logistic regression models. All variables included in the analysis were complete, with no missing data. Statistical significance was defined as *p* < 0.05.

## Results

### Cohort study

The findings from our institutional cohort are summarized in Table 1. Among 310 patients admitted with acute stroke, 8 patients (2.6%) developed in-hospital gout flares. All these patients presented with acute ischemic stroke; no cases of intracerebral hemorrhage were recorded among flare patients. Consequently, the rates of gout flares were 2.8% (8/285) vs. 0% (0/25) in patients with acute ischemic stroke and acute intracerebral hemorrhage in our cohort (*p* > 0.999, Fisher’s exact test). All patients were male, with a mean age of 67.5 (± 9.8) years. No gout flares were present on admission. Gout flares occurred at a mean of 4.1 (± 0.6) days after the index event and involved a non-paretic limb in 5 patients (62.5%), a paretic limb in 2 patients (25.0%), and both a paretic and non-paretic limb in 1 patient (12.5%). Four patients (50.0%) were newly diagnosed with hyperuricemia or gout during hospitalization. In-hospital delirium was recorded in 4 patients (50.0%) and aphasia in 1 (12.5%). All patients were treated with low-dose (0.5 mg qd) oral colchicine, and 5 (62.5%) additionally received intramuscular corticosteroids, with complete resolution of symptoms reported in all cases. The STROBE flowchart is provided in the Supplemental Material (Supplemental Figure S1).

**Table 1:** Demographic and clinical characteristics of patients with acute stroke and in-hospital gout flare from our institutional cohort

Patient Characteristics	Patients with Gout Flare (*n* = 8)	Patients without Gout Flare (*n* = 302)
**Age [mean (SD)]**	67.5 (9.84)	64.3 (12.7)
**Sex**,** n (%)**		
Male	8 (100%)	168 (56%)
**NIHSS on admission [median (IQR)]**	4 (2–5)	3 (1–7)
**Risk factors related to hyperuricemia**,** n (%)**		
Hypertension	2 (25%)	185 (61%)
Diabetes mellitus	1 (12.5%)	98 (32%)
Smoking	2 (25%)	163 (54%)
Atrial fibrillation	1 (12.5%)	27 (9%)
Dyslipidaemia	6 (75%)	154 (51%)
Alcohol	3 (37.5%)	17 (6%)
**Known untreated hyperuricemia / gout**,** n (%)**	1 (12.5%)	46 (15%)
**Known treated hyperuricemia / gout**,** n (%)**	3 (37.5%)	17 (6%)
Allopurinol	1 (12.5%)	12 (4%)
Febuxostat	2 (25%)	5 (2%)
**TOAST classification**,** n (%)**		
Large artery atherosclerosis	2 (25%)	27 (9%)
Small-vessel occlusion (lacune)	1 (12.5%)	76 (25%)
Cardioembolic	1 (12.5%)	35 (12%)
Stroke of undetermined etiology	4 (50%)	118 (39%)
Stroke of other determined etiology	0 (0%)	21 (7%)
**Intracerebral hemorrhage**,** n (%)**	0 (0%)	25 (8%)
**In-hospital delirium**,** n (%)**	4 (50%)	45 (15%)
**Aphasia**,** n (%)**	1 (12.5%)	55 (18%)
**rtPA**,** n (%)**	2 (25%)	42 (14%)
**Medication on admission**,** n (%)**		
Antiplatelets	1 (12.5%)	79 (26%)
Aspirin	1 (12.5%)	51 (17%)
Clopidogrel	0 (0%)	28 (9%)
Anticoagulants	0 (0%)	18 (6%)
Statin	3 (37.5%)	135 (45%)
Anti-hyperuricemia agents	3 (37.5%)	17 (6%)
**Mean time to gout flare**,** days [mean (+/-) SD]**	4.1 (+/- 0.6)	N/A
**Mean duration of hospitalization**,** days [mean (+/-) SD]**	8.6 (+/- 2.8)	8.5 (+/- 7.4)
**Newly diagnosed hyperuricemia / gout**,** n(%)**	4 (50%)	N/A
**Limbs affected**,** n (%)**		
Paretic	2 (25%)	N/A
Non-paretic	5 (62.5%)	N/A
Paretic and non-paretic	1 (12.5%)	N/A
**Flare treatment**,** n (%)**		
Colchicine	8 (100%)	N/A
Corticosteroids	5 (62.5%)	N/A

**Abbreviations**: AIS: Acute Ischemic Stroke; IQR: Interquartile Range; NIHSS: National Institutes of Health Stroke Scale; rtPA: Recombinant Tissue Plasminogen Activator; SD: Standard Deviation; TOAST: Trial of ORG 10,172 in Acute Stroke Treatment; N/A: not applicable

In logistic regression analysis, alcohol use was significantly associated with in-hospital gout flares (OR 10.38; 95% CI 2.50–43.14; *p* = 0.003). All flares occurred in men, indicating complete separation and a strong sex predilection; after Haldane-Anscombe correction, the odds ratio was 13.57 (95% CI 0.78–237.23; *p* = 0.074), approaching statistical significance though with wide CIs. None of the remaining variables met the threshold of statistical significance.

### Meta-analysis

#### Study selection and study characteristics

**Fig. 1:**
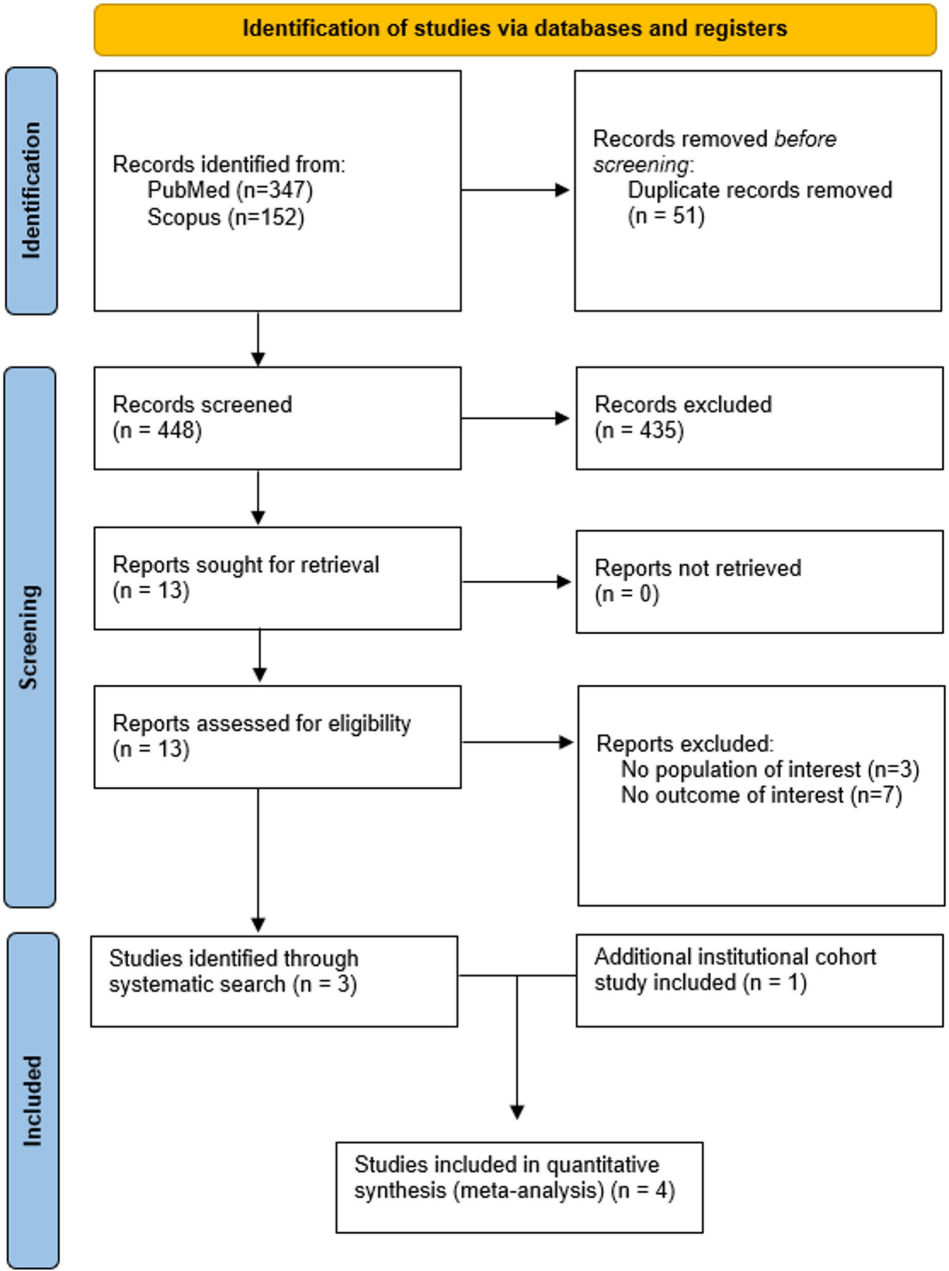
PRISMA flowchart diagram presenting the selection of eligible studies

The systematic search identified 499 records from PubMed (*n* = 347) and Scopus (*n* = 152), from which 51 duplicates were removed. Of the remaining 448 articles, 435 were excluded following title and abstract screening, and 13 underwent full-text assessment against the predefined inclusion and exclusion criteria. A total of 10 studies were subsequently excluded (Supplemental Table S1), resulting in three eligible studies, which were included along with our institutional cohort. In total, 13,722 patients with acute stroke were included in the meta-analysis (310 patients from our institutional cohort study and 13,412 from the three observational studies identified from the systematic literature search; detailed study characteristics are presented in Table 2) [32–34]. The PRISMA flowchart is presented in Fig. 1.

**Table 2:** Main characteristics of studies included in our meta-analysis

Study	Year	Country	Design	Population	Patients with stroke (*n*)	Patients with gout flare (*n*)
Chakravarty et al.[32]	1993	United Kingdom	Prospective observational cohort	Patients with acute stroke	111	4
Lin et al.[33]	2009	Taiwan	Retrospective observational cohort	Patients with acute ischemic stroke	920	60
Tan et al.[34]	2024	China	Retrospective observational cohort	Patients with acute stroke	12,381	372
Panagiotopoulos et al.	2025	Greece	Prospective observational cohort	Patients with acute stroke	310	8

#### Quality assessment and publication bias of included studies

The risk of bias of the included studies was evaluated using the Risk of Bias in Non-randomized Studies of Interventions (ROBINS-I) tool, with results presented in Supplemental Figures S2 and S3. Overall, the included studies were deemed to have a moderate risk of bias, as presented in the **Supplemental Material**.

Publication bias was not assessed due to the small number of included studies (< 10), which precludes reliable interpretation.

#### Primary and secondary outcomes

**Fig. 2:**
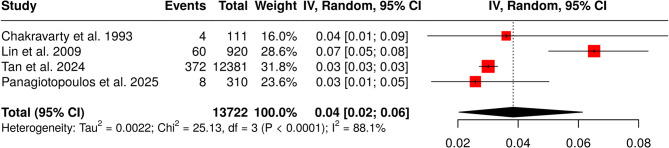
Forest plot presenting the pooled cumulative incidence of in-hospital gout flares among patients with acute stroke

**Fig. 3:**
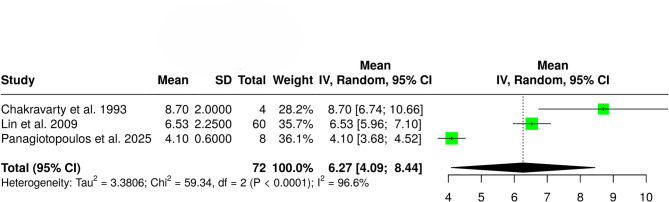
Forest plot presenting the pooled mean time from index stroke to flare onset

**Fig. 4:**
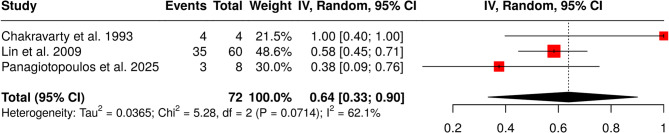
Forest plot presenting the pooled proportion of gout flares affecting the paretic limb in patients with acute stroke

The pooled results of the meta-analysis are summarized in Table 3. The pooled cumulative incidence of in-hospital gout flares among patients with acute stroke was 4% (95% CI: 2–6%; I² = 88.1%; p for Cochran Q < 0.01; Fig. 2). The pooled mean time from index stroke to flare onset was 6.27 days (95% CI: 4.09–8.44; I² = 96.6%; p for Cochran Q < 0.01; Fig. 3). Regarding localization, 64% of patients experienced flares in the paretic limb (95% CI: 33–90%; I² = 62.1%; p for Cochran Q = 0.07; Fig. 4).

**Table 3:** Overview of outcomes related to gout flares in acute stroke

Outcome	Studies (*n*)	Pooled Estimate	95% CI	I² (%)	*p*-Value for Cochran Q
Cumulative incidence of gout flares	4	0.04 (4%)	[0.02, 0.06]	88.1	< 0.01
Time to gout flare (days)	3	6.27 days	[4.09, 8.44]	96.6	< 0.01
Flares in paretic limb	3	0.64 (64%)	[0.33, 0.90]	62.1	0.07
Male sex	2	0.85 (85%)	[0.40, 1.00]	84.5	0.01
Ischemic stroke	2	0.97 (97%)	[0.94, 0.99]	0.0	0.70
First-time diagnosis of gout/hyperuricemia	2	0.61 (61%)	[0.48, 0.73]	0.0	0.53

**Abbreviations**: CI: Confidence Interval

#### Additional outcomes

Among patients who experienced gout flares, 85% were male (95% CI: 40–100%; I² = 84.5%; p for Cochran Q = 0.01; Supplemental Figure S4). Ischemic stroke was the predominant subtype in 97% of cases (95% CI: 94–99%; I² = 0%; p for Cochran Q = 0.70; Supplemental Figure S5). Data on stroke subtype were pooled from one study identified in the literature review [34], and from our institutional cohort, as the remaining studies included exclusively patients with ischemic stroke. A first-time diagnosis of hyperuricemia or gout was reported in 61% of patients (95% CI: 48–73%; I² = 0%; p for Cochran Q = 0.53; Supplemental Figure S6).

Acute gout flare management could not be meta-analyzed, as only in our institutional cohort gout flare treatments were recorded, with all patients (*n* = 8) treated with low-dose (0.5 mg qd) oral colchicine and 62.5% (*n* = 5) additionally receiving intramuscular corticosteroids. All treated patients experienced complete symptom resolution.

## Discussion

The present systematic review and meta-analysis, incorporating data from our single-center, prospective cohort documented the following findings. First, the pooled incidence of in-hospital gout flares among patients with acute stroke was 4%, a clinically significant rate that aligns with the previously reported prevalence of gout in stroke populations [1]. Second, most of the affected patients were male (85%) and presented with ischemic stroke (97%). Among the studies included in our systematic review, only Tan et al. stratified incidence by stroke subtype, reporting gout flares in 3.48% of ischemic stroke cases and 0.59% of hemorrhagic stroke cases, a statistically significant difference (*p* < 0.001) [34]. In our institutional cohort, no gout flares occurred among patients with intracerebral hemorrhage. Third, the onset of gout flares predominantly occurred during the first week following symptom onset (mean: 6.27 days), with a predilection for the paretic limb (64%). Fourth, over half of the patients (61%) who experienced a flare were newly diagnosed with hyperuricemia during hospitalization. Fifth, in our institutional cohort, 50% of patients with gout flare were diagnosed with in-hospital delirium, 12.5% with aphasia, and all were treated with colchicine with complete symptom resolution. In our cohort, in addition to male sex, alcohol consumption was identified as a significant risk factor for post-stroke gout flares.

To the best of our knowledge, this is the first meta-analysis to evaluate the incidence of gout flares in the acute stroke setting. Prior studies have primarily addressed the inverse association, wherein gout flares are linked to an increased risk of cerebrovascular and cardiovascular events. In a population-based study, patients with recent gout flares exhibited a significantly elevated risk of stroke and myocardial infarction within 0–60 days (adjusted OR, 1.93; 95% CI, 1.57–2.38) and 61–120 days (adjusted OR, 1.57; 95% CI, 1.26–1.96) following the flare episode [4]. In another study acute gout was associated with a higher 30-day risk of major adverse cardiovascular events (RR 1.70, 95% CI 1.41–2.04) [35]. This association has been attributed to NLRP3 inflammasome activation by urate crystals, initiating neutrophil recruitment, endothelial injury, and plaque destabilization [4]. Similar pathways have been implicated in large artery stroke, in which cholesterol crystals activate NLRP3, triggering IL-1β and IL-18 release and amplifying vascular inflammation [8]. These findings are consistent with a possible shared inflammatory axis linking acute gout and ischemic stroke. However, the temporal and causal direction of this association remains unclear. In our cohort, over half of patients had no prior diagnosis of gout or hyperuricemia, raising the possibility that subclinical hyperuricemia contributed to pre-stroke inflammation. In such cases, stroke-related physiological stress may unmask rather than precipitate acute gout.

The predominance of flares in the paretic limb also warrants consideration. Hemiparesis has been previously considered protective against joint inflammation, attributed to reduced mechanical loading and altered neuropeptide signaling, including decreased substance P and CGRP (calcitonin gene-related peptide) expression [36]. However, findings from our meta-analysis and recent studies suggest that the hemiplegic limb may instead be predisposed to gout flares, potentially due to impaired neurovascular regulation following stroke [37, 38]. Reduced mechanical stress [39], coupled with diminished peripheral vasoreactivity [40], could impair venous outflow, promote edema, and facilitate urate crystal deposition in the affected limb [33]. Central autonomic pathway disruption, involving mainly the hypothalamus and anterior cingulate cortex, could also compromise sympathetic control of peripheral vascular tone and predispose paretic limbs to edema formation, offering a plausible, yet largely speculative mechanism for the observed predisposition to gout flares [40]. Notably, the pathophysiological mechanisms outlined above should be considered as hypothesis-generating and warrant further investigation in larger multicenter cohorts.

The present findings underscore that early identification of gout flares in the post-stroke period requires systematic evaluation, including routine uric acid testing and targeted clinical assessment [33], particularly in patients with impaired communication. In our cohort, 50% of patients with flares developed in-hospital delirium and 12.5% had aphasia, limiting their ability to report pain. Sensory deficits in the affected limb may further delay recognition by attenuating nociceptive signaling.

Currently, no specific guidelines exist for managing gout flares in the context of acute stroke. The 2020 ACR guidelines recommend low-dose colchicine, NSAIDs (nonsteroidal anti-inflammatory drugs), or glucocorticoids as first-line therapies [41]. Colchicine inhibits microtubule polymerization, neutrophil activation, and NLRP3 inflammasome signaling, attenuating cytokine release and endothelial dysfunction -mechanisms relevant to vascular protection and plaque stabilization [8, 13, 14, 42]. Low-dose colchicine has been associated with reduced stroke recurrence and is increasingly considered for secondary prevention [12, 43], though benefits appear to be linked to prolonged use [12, 44]. When initiated for acute gout, extended colchicine therapy may offer additional vascular benefit. In our cohort, low-dose colchicine led to complete resolution of gout flares within 48 h, aligning with trials that show substantial improvement by 32–36 h in acute gout [45]. In contrast, cardiovascular prevention with colchicine appears to require sustained administration, with benefits reported during intermediate (31–150 days) and long-term (> 150 days) use [12, 46]. Glucocorticoids, by contrast, have been associated with increased 30-day mortality in ischemic and hemorrhagic stroke [47, 48], and NSAID-related stroke risk varies by agent, dose, and comorbidities [49]. Given its anti-inflammatory and cerebrovascular effects, colchicine may be the preferred treatment option for gout flares in the early post-stroke period.

Certain limitations of the present meta-analysis should be acknowledged. First, the small sample size of our institutional cohort limits statistical power and generalizability. Notably, while the meta-analysis demonstrated a higher frequency of flares in the paretic limb, flares in our cohort more commonly affected the non-paretic side - a finding likely attributable to sampling variation. Moreover, the limited number of hemorrhagic stroke cases, with no flares observed, limits subtype specific inferences and precludes meaningful comparisons between ischemic and hemorrhagic stroke, underscoring the need for further research to explore potential differences between stroke subtypes. Second, the observational design of both our cohort and the included studies introduces potential for selection bias and confounding. Third, our logistic regression analyses were based on a limited number of gout flare events [50], a fact that may increase the risk of model overfitting and both type I and type II errors. Fourth, the single-center nature of our cohort and the predominance of ischemic stroke constrain broader applicability, highlighting the need for larger, prospective multicenter studies. Fifth, as our cohort did not include long-term follow-up, potential associations between gout flares and recurrent vascular events warrant future exploration.

## Conclusions

In conclusion, gout flares occur approximately in one out of 25 acute stroke patients, especially in men presenting with acute ischemic stroke, typically within the first week following symptom onset and frequently involving the paretic limb. In over half of cases, the flare uncovers previously undiagnosed gout or hyperuricemia, emphasizing the need for systematic evaluation, particularly in patients with delirium or impaired communication, where diagnosis may be delayed. Early initiation of colchicine warrants consideration given its anti-inflammatory effects and potential to reduce stroke recurrence.

## Supplementary Information

Below is the link to the electronic supplementary material.

Supplementary Material 1

Supplementary Material 2

## Data Availability

All data needed to evaluate the conclusions in the paper are present in the main manuscript and in the supplemental material.

## Abbreviations

ACR: American College of Rheumatology
ACR/EULAR: American College of Rheumatology / European League Against Rheumatism
CI: Confidence Interval
CGRP: Calcitonin Gene–Related Peptide
NIHSS: National Institutes of Health Stroke Scale
PRISMA: Preferred Reporting Items for Systematic Reviews and Meta–Analyses
PROSPERO: International Prospective Register of Systematic Reviews
RCT: Randomized Controlled Trial
ROBINS: I–Risk of Bias in Non–randomized Studies of Interventions
rtPA: Recombinant Tissue Plasminogen Activator
