# Supplementary material for: Gout flares following acute stroke: a single-center cohort and a systematic review/meta-analysis
Source: Neurol Res Pract. 2025 Oct 15;7(1):75. doi: 10.1186/s42466-025-00424-w (PMC12529815; doi:10.1186/s42466-025-00424-w)

**SUPPLEMENTAL MATERIAL**

**Complete search algorithm used in MEDLINE (via PubMed) and Scopus database.**

**Supplemental tables:**

Supplemental Table S1. Table of excluded studies with reasons for exclusion.

**Supplemental Figures:**

Supplemental Figure S1. STROBE flowchart

Supplemental Figure S2. Traffic Light Plot presenting the quality assessment of included studies using the Risk Of Bias In Non-randomized Studies of Interventions (ROBINS-I) tool.

Supplemental Figure S3. Quality assessment of included studies using the Risk Of Bias In Non-randomized Studies of Interventions (ROBINS-I) tool, presented as percentages across all included studies.

Supplemental Figure S4. Forest plot presenting the pooled proportion of male patients with acute stroke experiencing in-hospital gout flares.

Supplemental Figure S5. Forest plot presenting the pooled proportion of ischemic stroke among patients with in-hospital gout flares.

Supplemental Figure S6. Forest plot presenting the pooled proportion of newly diagnosed hyperuricemia or gout among patients with in-hospital gout flares.

**ANALYSIS**

**Expanded Methods**

**Complete search algorithm used in MEDLINE (via PubMed) and Scopus database.**

The search string used in **PubMed** (all fields) was as follows: ("gout"[MeSH Terms] OR "gout"[All Fields] OR ("arthritis, gouty"[MeSH Terms] OR ("arthritis"[All Fields] AND "gouty"[All Fields]) OR "gouty arthritis"[All Fields] OR ("gouty"[All Fields] AND "arthritis"[All Fields])) OR ("uric"[All Fields] AND ("arthritis"[MeSH Terms] OR "arthritis"[All Fields] OR "arthritides"[All Fields] OR "polyarthritides"[All Fields])) OR (("gout"[MeSH Terms] OR "gout"[All Fields]) AND ("flare"[All Fields] OR "flares"[All Fields])) OR (("gout"[MeSH Terms] OR "gout"[All Fields]) AND ("attack"[All Fields] OR "attacked"[All Fields] OR "attacker"[All Fields] OR "attacker s"[All Fields] OR "attackers"[All Fields] OR "attacking"[All Fields] OR "attacks"[All Fields]))) AND ("stroke"[MeSH Terms] OR "stroke"[All Fields] OR "strokes"[All Fields] OR "stroke s"[All Fields])

The search algorithm in **Scopus** was the following: TITLE-ABS-KEY (gout OR uric AND arthritis OR gouty AND arthritis AND stroke)

**Supplemental Tables**

**Supplemental Table S1.** Table of excluded studies with reasons for exclusion.

| **Reason for exclusion** | **PMID (PubMed identifier)** |
| --- | --- |
| No population of interest | 35916846 |
| No population of interest | 39707504 |
| No population of interest | 9132325 |
| No outcome of interest | 28004164 |
| No outcome of interest | 39279144 |
| No outcome of interest | 31988479 |
| No outcome of interest | 37680887 |
| No outcome of interest | 35807064 |
| No outcome of interest | 24046469 |
| No outcome of interest | 36936597 |

**Supplemental Figures**

**Supplemental Figure S1**. STROBE flowchart

**
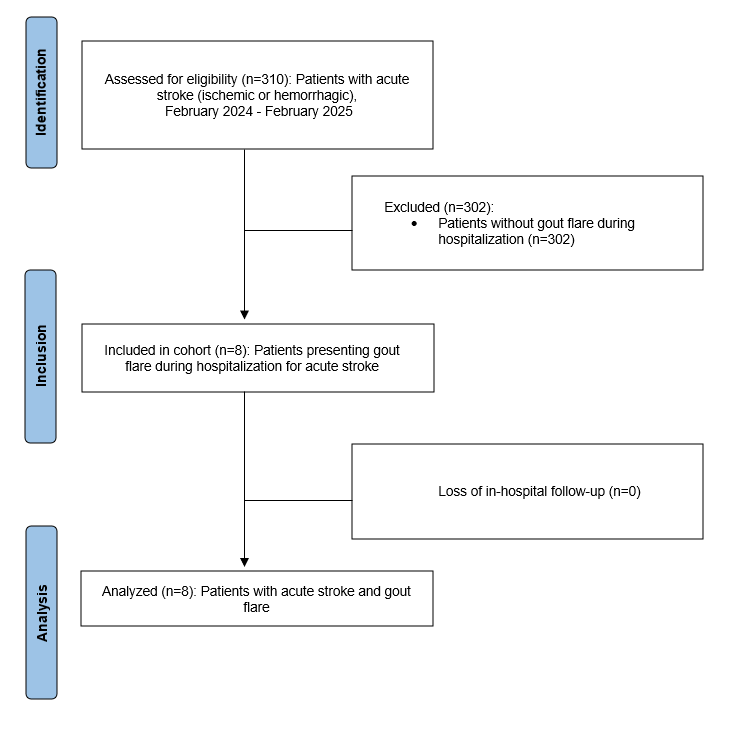
**

**Supplemental Figure S2.** Traffic Light Plot presenting the quality assessment of included studies using the Risk Of Bias In Non-randomized Studies of Interventions (ROBINS-I) tool.

 **Risk of bias domains**

**Supplemental Figure S3**. Quality assessment of included studies using the Risk Of Bias In Non-randomized Studies of Interventions (ROBINS-I) tool, presented as percentages across all included studies.

**
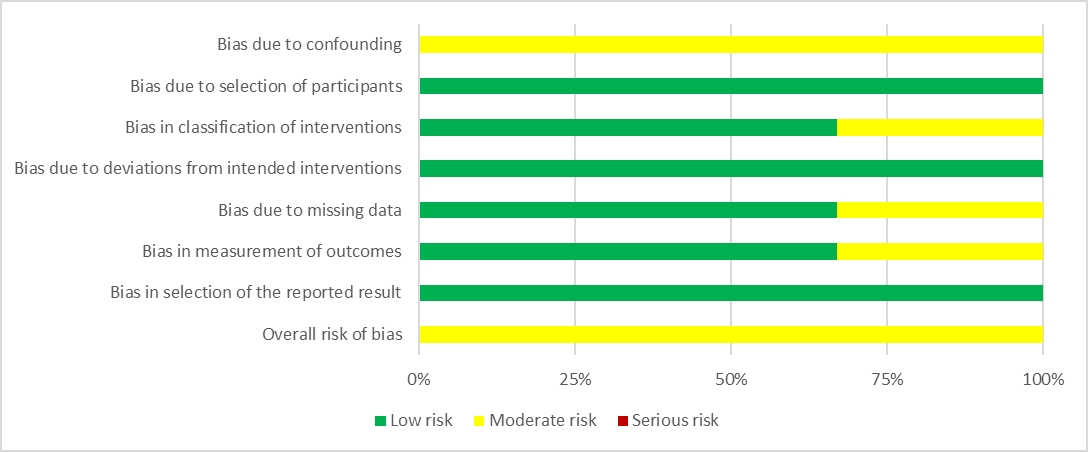
**


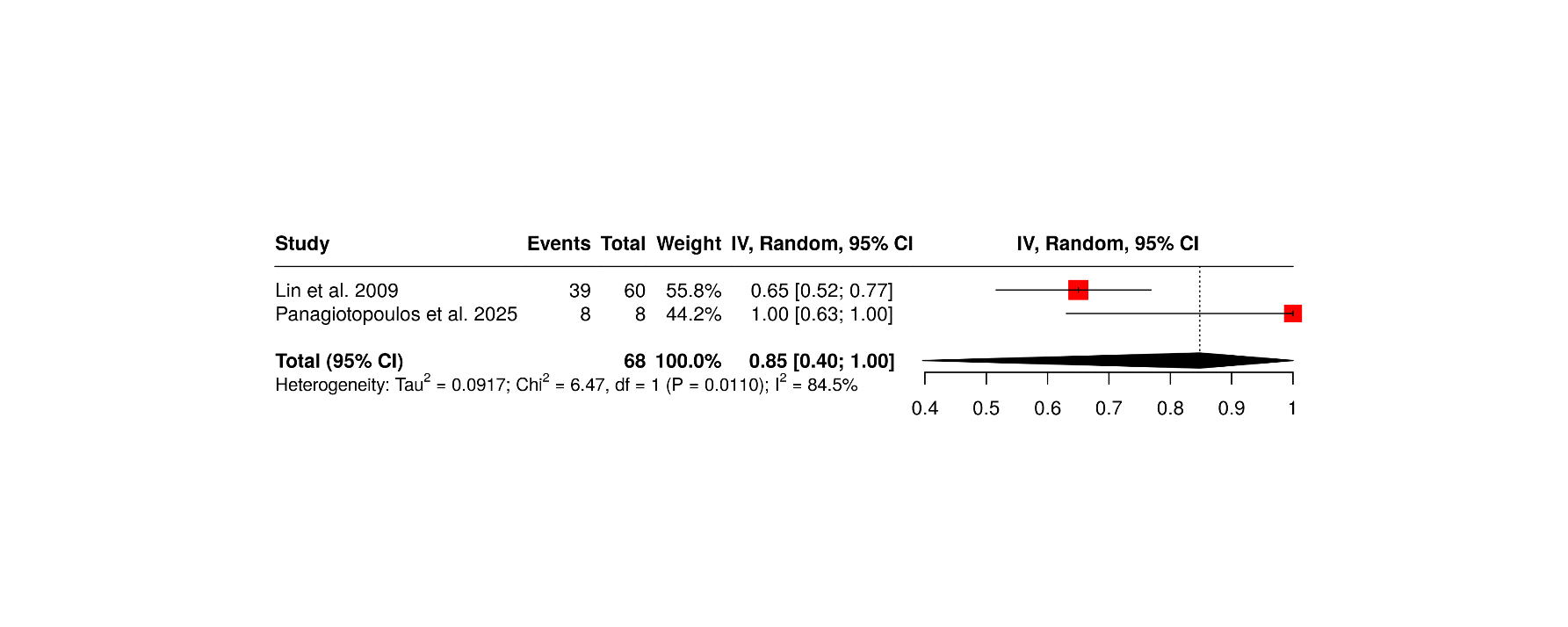
**Supplemental Figure S4.** Forest plot presenting the pooled proportion of male patients with acute stroke experiencing in-hospital gout flares.


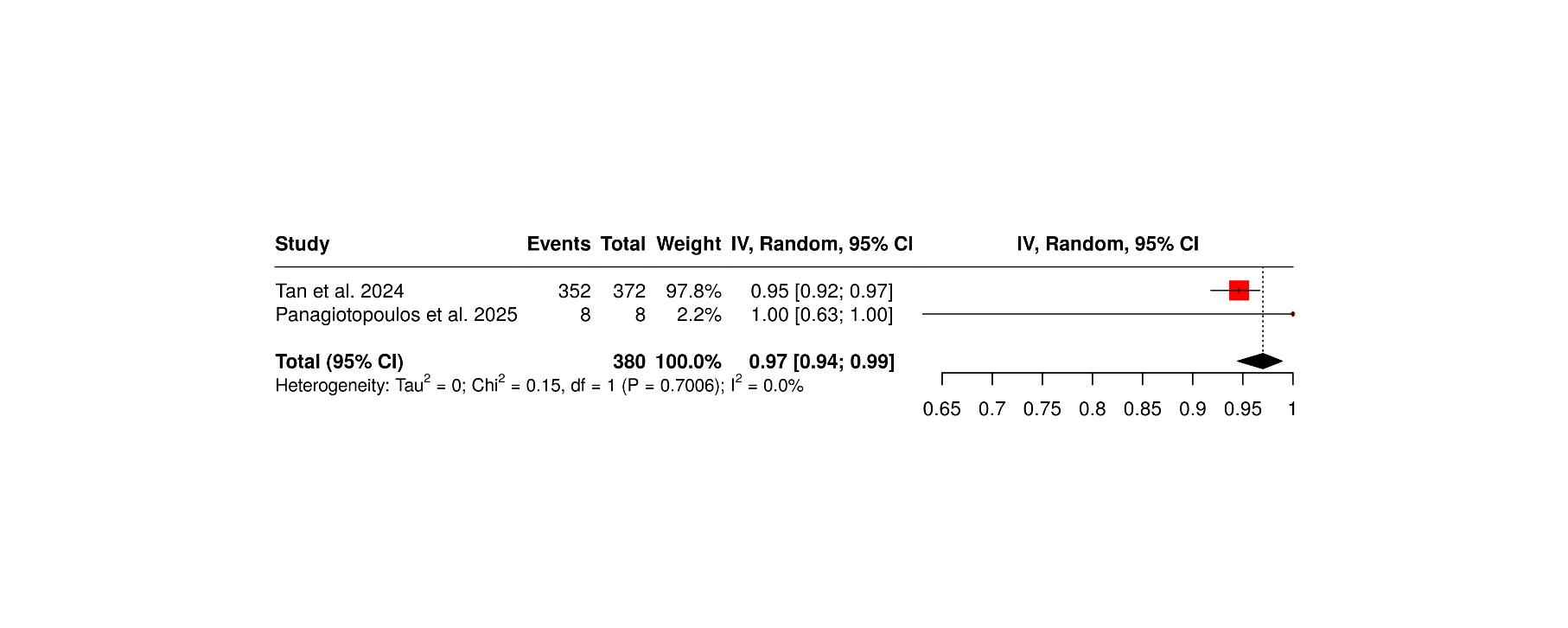
**Supplemental Figure S5**. Forest plot presenting the pooled proportion of ischemic stroke among patients with in-hospital gout flares.

**Supplemental Figure S6**. Forest plot presenting the pooled proportion of newly diagnosed hyperuricemia or gout among patients with in-hospital gout flares.


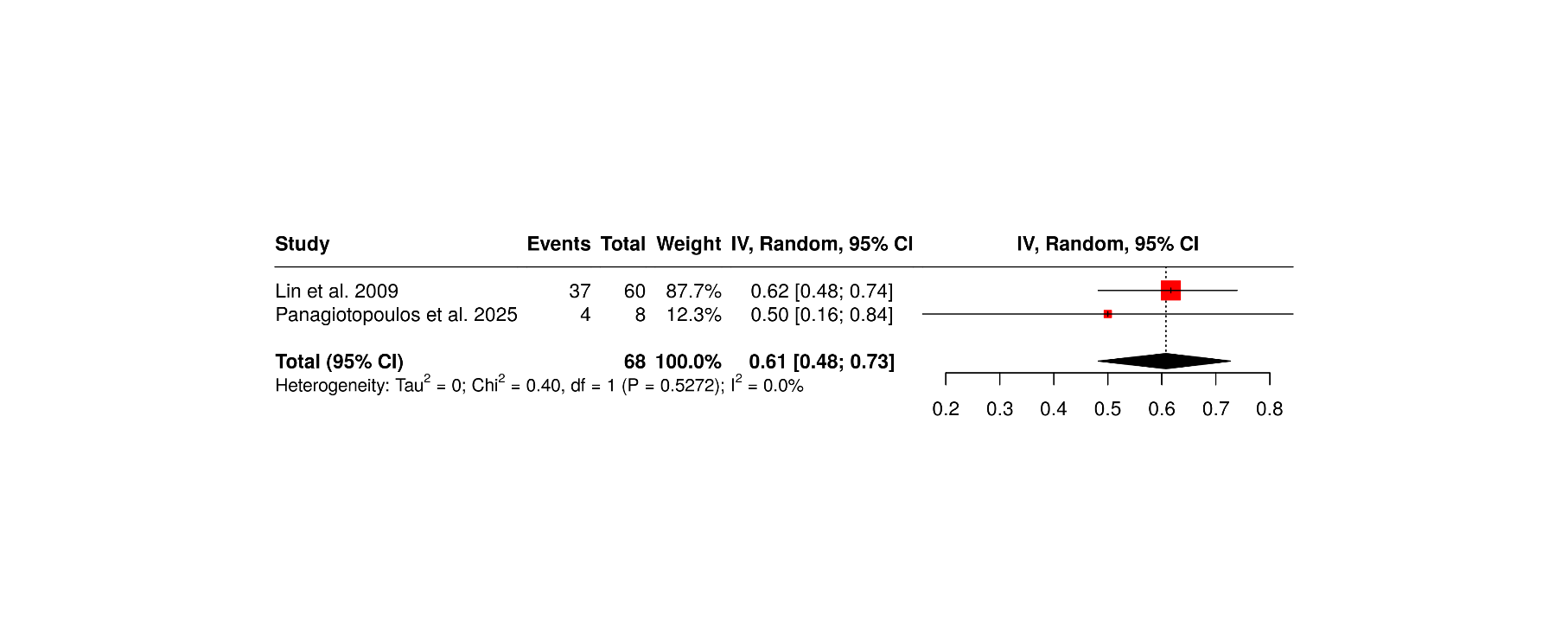

Supplement: Supplementary file 2 — Supplementary Material 2 [file 42466_2025_424_MOESM2_ESM.docx]
